# Supplementary material for: The association between upper gastrointestinal endoscopic findings and internal radiation exposure in residents living in areas affected by the Chernobyl nuclear accident
Source: PLoS One. 2022 Nov 30;17(11):e0278403. doi: 10.1371/journal.pone.0278403 (PMC9710756; doi:10.1371/journal.pone.0278403)
Supplement: Attachment — Submitted filename: Response to reviewers.doc [file pone.0278403.s003.doc]

August 18, 2022

Dear Reviewers,

Thank you for inviting us to submit a revised draft of our manuscript entitled, ‘Gastrointestinal findings in residents living in areas affected by the Chernobyl nuclear accident’ [ms. no PONE-D-22-16737] to *PLoS ONE*. We also appreciate the time and effort you and each of the reviewers have dedicated to providing insightful feedback on ways to strengthen our paper. Thus, it is with great pleasure that we resubmit our article for further consideration. We have incorporated the changes that reflect the detailed suggestions you have so graciously provided. We also hope that our edits and the responses we provide below satisfactorily address all the issues and concerns you and the reviewers have noted.

First of all, we apologize that eight minors were included in our study due to a mistake in age calculation, who do not meet the criteria for participation in our study. We excluded them from our analyses and performed all the calculations and evaluations again. However, the findings and conclusions of our study did not change and remain the same.

To facilitate your review of our revisions, the following is a point-by-point response to the questions and comments delivered in your E-mail dated 13 July 2022. Also, we had native speakers of English proofread our English writing again.

- **Editor**

**Comment 1.** Please ensure that your manuscript meets PLOS ONE’s style requirements, including those for file naming.

**Response.** We have ensured that the manuscript meets all the PLOS ONE’s style requirements, including those for file naming.

**Comment 2.** Please include a complete copy of PLOS ONE’s ‘questionnaire on inclusivity’ in global research in your revised manuscript. Please upload a completed version of your questionnaire as Supporting Information when you submit your manuscript.

**Response.** We have completed the questionnaire on inclusivity and will upload it when we submit our revised manuscript as recommended.

**Comment 3.** Please clarify how participants were recruited and how they were provided information about and given access to the opt-out consent.

**Response.** We apologize for the lack of clarity. We re-examined all the documents issued by the University Ethics Committee and ensured that all consent forms were submitted in writing. Accordingly, we have revised the section on participant recruitment and elucidated the process of providing and receiving written informed consent.

(Line 142-151 of clean copy)

*All of them were under the medical surveillance of the Zhytomyr Inter-Area Medical Diagnostic Center (Medical Center) that provided medical services for residents from our research area. We invited all patients who sought medical assistance in the Medical Center for any GI symptoms or digestive organ disorders during the study period to participate in the study. Residency registration within the research area at the moment of examination was mandatory. Those who agreed to participate in the study, initially received a detailed description of the process and content of the study, and were then asked to provide written consent. Additionally, they filled out a questionnaire regarding their lifestyle and diet habits and underwent gastrointestinal endoscopy and measurement of their internal body burden on the Whole Body-Counter (WBC).*

(Line 185-187 of clean copy)

*Informed consent was obtained from each individual through the written form, which indicated agreement for participation in the research.*

**Comment 4.** Please provide additional details regarding participants consent. In the ethics statement in the Methods and online submission information, please ensure that you have specified what type you obtained (for instance, written or verbal, and if verbal, how it was documented and witnessed). If your study includes minors, state whether you obtained consent form parents of guardians. If the need for consent was waived by the ethics committee, please include this information.

**Response.** We have revised the section concerning obtaining consent and elucidated the process of providing information and receiving consent in the Methods. Please refer to line 142-151 and to line 185-187 regarding consent from minors (shown above in Comment 3).

As for minor participants, we discovered that eight minors were included in our study due to a mistake in age calculation, who do not meet with the criteria for participation in our study. Consequently, we excluded them from our analyses and performed all the calculations and evaluations again with the remaining participants. In general, the overall results and findings of our study did not change, and the study conclusions remain the same.

**Comment 5.** We note that you have indicated that data from this study are available upon request. PLOS only allows data to be available upon request if there are legal or ethical restrictions on sharing data publicly.

a) If there are ethical or legal restrictions on sharing de-identified data set, please explain them in detail and who has imposed them. Please also provide contact information for data access.
b) If there are no restrictions, please upload minimal anonymized data set necessary to replicate your study findings as either Supporting Information files or to a stable, public repository and provide with relevant URLs, DOIs, or accession numbers. Please see guidelines on how to de-identify and prepare clinical data for publication.

**Response.** We have provided bare minimal anonymised data to enable replication and reproduction of the findings reported in our paper.

**Comment 6.** We note that you have included the phrase “data not shown” in your manuscript. Unfortunately, this does not meet our data sharing requirements. PLOS does not permit reference to inaccessible data. If the data are not a core part of the research being presented in your study, we ask that you remove the phrase that refers to these data.

**Response.** We have removed the phrase “data not shown” and ensured that all numbers and results can be reproduced using the provided data.

- **Reviewer #1:**

We are grateful to reviewer #1 for the critical comments and useful suggestions that have helped us improve our paper considerably. As indicated in the responses that follow, we have considered all these comments and suggestions while revising our paper.

**Comment 1.** Fig.3: There is no X-axis label

**Response.** We have edited the part of Figure 3 that was commented on and have labelled the X-axis.

**Comment 2.** Do the authors take into account the effect of external radiation exposure.

**Response.** Our main research target was only low-dose internal exposure from ^137^Cs and GI findings, and therefore, we did not account for external exposure.

(Line 301-308 of clean copy)

*Internal exposure from ^137^Cs, as opposed to dose rates from external exposure, decreases more slowly in the general population and its contribution to total body exposure increases gradually [12]. Therefore, our study attempts to identify the potential effect of low internal doses of ^137^Cs on the digestive organs of the human body, as it is known that ^137^Cs accumulates in muscles and visceral organs. In Semoshkina et al’s study, it was reported that ^137^Cs was highly transferred to the spleen, lungs, heart, muscles, kidneys, skin and bones in horse tissue taken 90 days after the beginning of radionucllide administration [13].*

**Comment 3.** Why ^137^Cs was chosen? It is possible, that ^90^Sr, having almost identical radioactive half-life, but totally different biological half-life, can also contribute to formation of gastrointestinal findings. Was the impact of present eating habits of people excluded.

**Response.** We have amended the first paragraph of the Discussion section, where we elucidate our reason for choosing ^137^Cs as the primary interest of our study, as opposed to another radionuclides.

(Line 296-308 of clean copy)

*In this study, the key focus was internal exposure emitted from ^137^Cs and its potential effects on the gastrointestinal system of the human body. A majority of studies concerning the CNPP accident that investigate the effects of radiation on the human body have concentrated their primary interests in ^137^Cs and internal exposure. ^137^Cs has a greater effect on people than other radionuclides due to its properties, such as a long half-life, amount released into environment and dispersion in a wider area. Internal exposure from ^137^Cs, as opposed to dose rates from external exposure, decreases more slowly in the general population and its contribution to total body exposure increases gradually [12]. Therefore, our study attempts to identify the potential effect of low internal doses of ^137^Cs on the digestive organs of the human body, as it is known that ^137^Cs accumulates in muscles and visceral organs. In Semoshkina et al’s study, it was reported that ^137^Cs was highly transferred to the spleen, lungs, heart, muscles, kidneys, skin and bones in horse tissue taken 90 days after the beginning of radionucllide administration [13].*

The impact of eating habits was not excluded in our study.

(Line 308-311 of clean copy)

*We assume that a majority of the residents have simillar patterns and frequencies of internal exposure from ^137^Cs in the body, which continuously varies over time. This assumption is based on as earlier 10-year study from the same area [14].*

**Comment 4.** Have all people in the cohort lived in the area all the time since the accident? Was the migration excluded.

**Response.** We added some additional information confirming that all participants only needed residency registration within the study area at the time of examination.

(Line 144-147 of clean copy)

*We invited all patients who sought medical assistance in the Medical Center for any GI symptoms or digestive organ disorders during the study period to participate in the study. Residency registration within the research area at the moment of examination was mandatory.*

- **Reviewer #2:**

We are grateful to reviewer #2 for the critical comments and useful suggestions that have helped us improve our paper considerably. As indicated in the responses that follow, we have considered all these comments and suggestions while revising our paper.

- **Reviewer #3:**

We are grateful to reviewer #2 for the critical comments and useful suggestions that have helped us improve our paper considerably. As indicated in the responses that follow, we have considered all these comments and suggestions while revising our paper.

**Comment 1.** I suggest performing additional calculations. Multivariate regression for men and women separately might be able to reveal an interaction between alcohol consumption and wild food intake, when we assume that alcohol consumption is lower in women compared to men.

**Response.** We have performed the suggested analysis and added its results to the Results section.

(Line 289-294 of clean copy)

*We also performed regression analysis for women separately, as it could reveal an interaction between alcohol consumption and wild food intake, assuming that alcohol consumption is lower in women compared to men. Regression analysis for the only-women group revealed results similar to Table 3, indicating significance for age, alcohol consumption, intake of wild food (p<0.05), while smoking and the level of Bq/kg seemingly did not affect the number of GI findings (p>0.05).*

**Comment 2.** Figure 3: “detectable” and “detected” are used in confusion. Please apply the term consistently. I suggest using “patients with WBC counts of Cs137 above the detection limit”.

**Response.**

We have edited the specified sentence in accordance with your suggestion as shown in line 257-258 “participants with ^137^Cs exposure above the WBC’s detection limit”. We have also revised “detectable” to “detected” in Figure 3 for consistency.

(Line 262-263 of clean copy)

*Fig 3 demonstrates the average Bq/kg and percentage of participants with ^137^Cs exposure above the WBC’s detection limit for each number of GI findings in the group.*

- **Reviewer #5:**

We are grateful to reviewer #5 for the critical comments and useful suggestions that have helped us improve our paper considerably. As indicated in the responses that follow, we have considered all these comments and suggestions while revising our paper.

**Comment 1.** It is pity that there is no comparison with a control group, i.e. population non-exposed to Cs-137 and/or not intentionally looking for a medical examination and/or alcohol non-consumers/smokers. These facts significantly decrease the value of the study.

**Response.** We agree with your comment and have accordingly mentioned these imperfections in the limitations.

**Comment 2.** What was the MDA value in Bq/kg of bodyweight (e.g. 270 Bq per body vs average 6.2±11.8 Bq/kg vs. detectable all 20.6 Bq/kg).

**Response.** The MDA value of the Whole-Body Counter we used during our study was 270 Ber per body. The average Bq/kg for the entire population was 6.2±11.8 whereas the average Bq/kg for the group with exposure above the Whole-Body Counter’s detection limit was 20.6. We have additionally edited the titles of rows in Table 2 for clarity.

**Comment 3.** I suggest evaluating and estimating the long-term doses to the GI tract. Instead of correlation analysis between the age, WBC detected activity and GI findings, it would be more interesting to try to estimate the integral lifetime low-dose based on WBC data and a questionnaire, possibly monitoring subject habits, place of living and potential long-term exposure.

**Response.**

We agree that long-term doses and lifetime exposure would be of greatly interest to research. However, our study is not designed to follow-up with affected individuals and monitor participant habits and place of living. We have added these points to the limitations.

(Line 429-433 of clean copy)

*We did not conduct follow-ups with the participants any longer research period or estimate their life-long accumulated low-dose radiation. Additionally, we also did not assess the effect of life-long radiation on the increase of GI findings. However, we admit that these points should be considered in future studies and included in research methods.*

**Comment 4.** Data of the study were not provided due to personal data protection – however some anonymised data could be possibly provided in some shortened summarized form.

**Response.**

We have provided anonymised data to enable replication and reproduction of the study findings.

With the incorporation of these amendments to our final manuscript, we hereby resubmit our manuscript for a secondary evaluation. We hope that the revised version of our paper is now suitable for publication in *PLoS ONE*, and we look forward to hearing from you at your earliest convenience.

Sincerely,

Naomi Hayashida, M.D., PhD.

Professor of Division of Strategic Collaborative Research, Center for Promotion of Collaborative Research on Radiation and Environment Health Effects, Atomic Bomb Disease Institute, Nagasaki University

1-12-4 Sakamoto, Nagasaki 852-8523, JAPAN

TEL: +81-95-819-8507

FAX: +81-95-819-8508

E-mail: [naomin@nagasaki-u.ac.jp](mailto:naomin@nagasaki-u.ac.jp)
