# Supplementary material for: The association between upper gastrointestinal endoscopic findings and internal radiation exposure in residents living in areas affected by the Chernobyl nuclear accident
Source: PLoS One. 2022 Nov 30;17(11):e0278403. doi: 10.1371/journal.pone.0278403 (PMC9710756; doi:10.1371/journal.pone.0278403)
Supplement: Attachment — Submitted filename: Response to reviewers.doc [file pone.0278403.s004.doc]

September 8, 2022

Dear Reviewer #4,

Thank you for inviting us to submit a revised draft of our manuscript entitled, ‘Gastrointestinal findings in residents living in areas affected by the Chernobyl nuclear accident’ [ms. no PONE-D-22-16737R1] to *PLoS ONE*. We also appreciate the time and effort you have dedicated to providing insightful feedback on ways to strengthen our paper. Thus, it is with great pleasure that we resubmit our article for further consideration. We have incorporated the changes that reflect the detailed suggestions you have so graciously provided. We also hope that our edits and the responses we provide below satisfactorily address all the issues and concerns you have noted.

**Comment 1.** I recommended that the authors should extend their regression analysis. Has this been done?

**Response.** We have performed the suggested analysis and added its results to the Results section.

(Line 289-294 of clean copy)

*We also performed regression analysis for women separately, as it could reveal an interaction between alcohol consumption and wild food intake, assuming that alcohol consumption is lower in women compared to men. Regression analysis for the only-women group revealed results similar to Table 3, indicating significance for age, alcohol consumption, intake of wild food (p<0.05), while smoking and the level of Bq/kg seemingly did not affect the number of GI findings (p>0.05).*

With the incorporation of these amendments to our final manuscript, we hereby resubmit our manuscript for a secondary evaluation. We hope that the revised version of our paper is now suitable for publication in *PLoS ONE*, and we look forward to hearing from you at your earliest convenience.

Sincerely,

Naomi Hayashida, M.D., PhD.

Professor of Division of Strategic Collaborative Research, Center for Promotion of Collaborative Research on Radiation and Environment Health Effects, Atomic Bomb Disease Institute, Nagasaki University

1-12-4 Sakamoto, Nagasaki 852-8523, JAPAN

TEL: +81-95-819-8507

FAX: +81-95-819-8508

E-mail: [naomin@nagasaki-u.ac.jp](mailto:naomin@nagasaki-u.ac.jp)
