# Supplementary material for: The association between upper gastrointestinal endoscopic findings and internal radiation exposure in residents living in areas affected by the Chernobyl nuclear accident
Source: PLoS One. 2022 Nov 30;17(11):e0278403. doi: 10.1371/journal.pone.0278403 (PMC9710756; doi:10.1371/journal.pone.0278403)
Supplement: Attachment — Submitted filename: academic_editor_comments_PONE-D-22-16737R2.docx [file pone.0278403.s005.docx]

Academic editor:

Title- The title is confusing. The “Gastrointestinal finding” looks like a hanging title. Gastrointestinal cancer incidence? Gastrointestinal diseases? Gastrointestinal symptoms? Please revise the title, it must be concise and reflecting your work, not too general.

Please define the GI findings. Are they non-cancer disease? Lien 145-146 give a glace of definition GI finding, but I believe it is useful in abstract and introduction.

Abst, Line 24-25 – There is no concrete evident that all the locals are digesting the contaminated Cs-137 food stuffs. The author should be careful with the statement unless there is a report from WHO, ICRP epidemiological studies that show the significant evidence from important Cs biomarkers eg faeces and bone etc. The first line 23-24 also should be revised. Externally exposures from gammas from Cs-137 are true but not for internal exposures of the all the local peoples considering biological half lives of Cs-137 in few tenth days.

Abst, methodology – Please reorganise the method and approaches used in this study. The abstract writing is very important component to highlight background, problem statement, methodology, result and discussion and mostly importantly the conclusion. In your case, the methods and approach are not in a good order.

- You can just combine and make it concise. All the data on internal 137Cs concentration and GI findings in people were collected from 2016 to 2018 in the Zhytomyr region, Ukraine. The Whole-Body Counter and Gastro Endoscope were used to detect outcomes in the study participants.
- What are your main data inputs? Cs-137 and GI symptoms? Each data inputs have parameters of score/merits to see the correlation? eg. intake of wild forest food, smoking habits, and alcohol consumption.
- How many main data collected and how many secondary data (questionnaires) are obtained? How many participates in WBC and GI endoscope?

Abst, result/discussion – limited results are given. The scientific values are important but not included in abstract? Range and average internal accumulated Bq are detected in GI system. Any finding from gastro endoscope? You mentioned the alcohol is a major cause for GI finding, yet no statistical hypothesis test to assess the likelihood of alcohol impacts on GI findings using analysis of variance, or the non-parametric test eg Welch’s anova or Kruskal Wallis test etc. All the parameters that affecting the GI finding eg. sex, intake of wild forest foodstuff, and alcohol consumption need to be assessed too. Line 175-180 gives the details, but found none in abstract

a) -***However, the proportion of a higher number of GI findings increased with the age of the groups. This tendency can be seen in the proportions of the 3 and 4 GI findings****. However, the proportion of* ***5 GI findings was detected only in two age groups, namely 51–60 years and 61–70 years****. We also assessed the significance of the average Bq/kg in different age groups, which* ***turned out to have no significant difference (p=0.461).*** ***The highets average Bq/kg was observed in the group of 81-83 years old****. However this group may not be representative, as it only containes 6 participants. If this group is excluded,* ***the younger the age, the higher the level of internal radiation exposure.***
b) ***Factors such as Bq/kg and smoking did not affect the increase of number of GI findings.***
c) ***age, sex, intake of wild food, and alcohol consumption contribute significantly to the increase in the number of GI findings.***
d) ***They showed significant differences (p<0.05), suggesting that the increased levels of GI findings in detectable groups are more likely to have originated due to alcohol consumption.***

The highlighted inputs above should be included in abstract.

Abst, results – “Other factors, like the average level of a becquerel per kilogram of body mass (Bq/kg) and smoking”. Kindly revised the statement. Its confusing. What activity is this? Cs-137? The for the smoking what is the tangible data ? how do you compare that?

Abst, conclusion – no conclusion have been drawn.

Abst, recommendation - My opinions is that you draw a conclusion from your 2 years of work before suggesting a study that efficient in proving you hypothesis from this study.

Intro, line 55-60 – instead of stating the existence of BEIR VI’s comprehensive report on effect off low dose, it is more appropriate if the author highlighting the BEIR, IAEA, UNSCEAR and WHO findings on Cs-137 contaminated foodstuffs in Chernobyl.

Intro, 61-65 – “highest exposed recovery workers”. Kindly check the suitability to include this information because it may be a dominated by major gamma external exposures. The written introductions are lack of focus. The author highlights the “recovery workers” which out of the topic according to this work aims of motivation and title. It is necessary up to these points, the authors only addressing the literature work of investigation of Cs-137/other isotopes intakes by the Chernobyl’s publics.

Intro, 66-75 –the whole paragraph just highlighting the finding for the thyroid cases from ingested iodine contaminated food. Can you make some hypothesis of these finding in relation to your work GI findings based for Cs-137 contamination? At least a line stating that increased I-131 thyroid cases after few years of the incident shows that high possibility of unavoidable Cs-137 ingestion through local foodstuffs.

Line 76-116 –These para(s) highlighting the BEIR VI findings of non-cancer risks based on LSS & abs data from UNSCEAR reports in your work. Discussing the background is necessary however you include broaden scopes of internal organ non-cancer incidences. This manuscript will be impactful to readers if you narrow the scopes by focusing the literature works/background studies on GI impacts from low dose or Cs-137 ingestions.

Line 124-126 - The statement need citations.

Line 163-164 - *We used the number of GI findings detected in each participant to indicate the extent of gastrointestinal lesions.* What number? A score? How do you scale it? 1 until 5?..please elaborate after the line.

Line 163 – Please elaborate the criteria for finding 1 – 5? Extent of lesion and condition?

Line 166 – radiation? Or activity levels?

Line 170 – cleaned? Filtered?

Methodology – Please include the work flowcharts of your work. From fill form, wbc screening – GI endoscopy, classification 0 Bq or higher, data treatment, statistical tes etc.

Table 1 - P-value of what significance test? Men vs women?

Table 1 – Define body burden and detected subject

Result – Based on highlighted lines below, kindly explain how do you treat the difference sizes of population groups in the Mann Whitney U test? How to assure no bias output from the analysis? P=0.461 value may be affected by the significant difference of group size?

*We also assessed the significance of the average Bq/kg in different age groups, which turned out to have no significant difference (p=0.461). The highets average Bq/kg was observed in the group of 81-83 years old.*

Line 279 - dependent variables? Not independent?

Line 296-326 – redundant as intro and methodology. Please remove. Discussion is only to discuss your inputs and highlight the finding which started at line 327.
