# Supplementary material for: The association between upper gastrointestinal endoscopic findings and internal radiation exposure in residents living in areas affected by the Chernobyl nuclear accident
Source: PLoS One. 2022 Nov 30;17(11):e0278403. doi: 10.1371/journal.pone.0278403 (PMC9710756; doi:10.1371/journal.pone.0278403)
Supplement: Attachment — Submitted filename: Response_to_Editor.docx [file pone.0278403.s006.docx]

November 8, 2022

Mohamad Syazwan Mohd Sanusi, PhD

Academic Editor

PLOS ONE

Dear Dr. Sanusi,

Thank you for inviting us to resubmit a revised draft of our manuscript entitled, ‘Gastrointestinal findings in residents living in areas affected by the Chernobyl nuclear accident’ [PONE-D-22-16737R2] to *PLoS ONE*.

We appreciate your earnest comments and valuable suggestions that have notably improved the structure and alleviated the comprehension of our manuscript.

We have incorporated the changes in the manuscript to reflect the detailed suggestions you have so graciously provided. We also hope that our edits and the responses we provide below satisfactorily address all the issues and concerns.

To facilitate your review of our revisions, below are point-by-point responses to the questions and comments delivered via your E-mail dated September 30, 2022.

We have considered all the comments and suggestions that were submitted and responded as provided below.

**Comment 1.**

Please review your reference list to ensure that it is complete and correct. If you have cited papers that have been retracted, please include the rationale for doing so in the manuscript text, or remove these references and replace them with relevant current references. Any changes to the reference list should be mentioned in the rebuttal letter that accompanies your revised manuscript. If you need to cite a retracted article, indicate the article’s retracted status in the References list and also include a citation and full reference for the retraction notice.

**Response.**

We have ensured that the reference list in the manuscript is complete and correct. We did not find a record of retraction for any paper we have included in the reference list and our manuscript.

**Comment 2.**

Title- The title is confusing. The “Gastrointestinal finding” looks like a hanging title. Gastrointestinal cancer incidence? Gastrointestinal diseases? Gastrointestinal symptoms? Please revise the title, it must be concise and reflecting your work, not too general.

**Response.**

We changed the title of our paper to “**The association between upper gastrointestinal endoscopic findings and internal radiation exposure in residents living in areas affected by the Chernobyl nuclear accident**”.

**Comment 3-1.**

Please define the GI findings. Are they non-cancer disease?

**Response.**

The GI findings we investigated in this study included all types of upper GI endoscopic diagnoses that can be diagnosed by a gastroenterologist according to the International Classification of Disease. We use the word “diagnosis” to distinguish from “findings.” “Upper GI endoscopic diagnosis” represents each finding such as gastritis, duodenitis, duodenogastric reflux, gastroesophageal reflux, etc.

**Comment 3-2.**

Line 145-146 give a glace of definition GI finding, but I believe it is useful in abstract and introduction.

**Response.**

We added details regarding the definition of GI findings in the introduction. Adding more details to the abstract seems difficult as the journal puts limitation of 300 words. We use the word “diagnosis” to distinguish from “findings.” “Upper GI endoscopic diagnosis” represents each finding such as gastritis, duodenitis, duodenogastric reflux, gastroesophageal reflux, etc.

We amended Introduction section according to your recommendation.

(Line 121-126 of clean copy)

*The findings of upper GI endoscopic examination were interpreted according to the International Classification of Diseases (ICD) of World Health Organization (WHO). Common upper GI endoscopic diagnoses such as gastritis, duodenitis, duodenogastric reflux, gastroesophageal reflux, stomach ulcer, diaphragmatic hernia, and other diagnoses, including a few cases of cancer, were diagnosed in a wide range of combinations among most of the screened participants.*

**Comment 4.**

Abstract, Line 24-25 – There is no concrete evident that all the locals are digesting the contaminated Cs-137 food stuffs. The author should be careful with the statement unless there is a report from WHO, ICRP epidemiological studies that show the significant evidence from important Cs biomarkers eg faeces and bone etc. The first line 23-24 also should be revised. Externally exposures from gammas from Cs-137 are true but not for internal exposures of the all the local peoples considering biological half-lives of Cs-137 in few tenth days.

**Response.**

We revised corresponding sentences according to your recommendation.

(Line 22-26 of clean copy)

*Many people living around the Chernobyl Nuclear Power Plant (CNPP) have been exposed to ^137^Cs for several decades after the CNPP accident. Although half-life of ^137^Cs is about 30 years, some wild forest foodstuffs are contaminated by ^137^Cs even now. We pointed out in a previous report that low-dose internal radiation has been occasionally detected in people’s body.*

**Comment 5.**

Abst, methodology – Please reorganise the method and approaches used in this study. The abstract writing is very important component to highlight background, problem statement, methodology, result and discussion and mostly importantly the conclusion. In your case, the methods and approach are not in a good order.

-	You can just combine and make it concise. All the data on internal ^137^Cs concentration and GI findings in people were collected from 2016 to 2018 in the Zhytomyr region, Ukraine. The Whole-Body Counter and Gastro Endoscope were used to detect outcomes in the study participants.

-	What are your main data inputs? Cs-137 and GI symptoms? Each data inputs have parameters of score/merits to see the correlation? eg. intake of wild forest food, smoking habits, and alcohol consumption.

-	How many main data collected and how many secondary data (questionnaires) are obtained? How many participates in WBC and GI endoscope?

**Response.**

We added and modified the order of the sentences for more clarity and consistency. As the guidelines of PLOS ONE for the abstract are substantially limited, we amended the Abstract concisely and elaborated Materials and methods and Results sections of the manuscript according to your suggestions.

(Line 30-38 of clean copy)

*Overall, 1,612 residents were assessed for internal ^137^Cs concentration using Whole-Body Counter and their digestive organs were screened with upper GI endoscopy from 2016-2018 in the Zhytomyr region, Ukraine. All participants answered to the questionnaire including their background, intake of wild forest foodstuff, intake frequency, smoking habits, and alcohol consumption. We checked the number of upper GI endoscopic diagnosis per person to assess the extent of damage to the upper digestive organs. Next, we statistically analyzed associations between this number and age, sex, level of internal exposure dose, alcohol consumption, wild forest foodstuff intake, and smoking.*

**Comment 6.**

Abst, result/discussion – limited results are given. The scientific values are important but not included in abstract? Range and average internal accumulated Bq are detected in GI system. Any finding from gastro endoscope? You mentioned the alcohol is a major cause for GI finding, yet no statistical hypothesis test to assess the likelihood of alcohol impacts on GI findings using analysis of variance, or the non-parametric test eg Welch’s anova or Kruskal Wallis test etc. All the parameters that affecting the GI finding eg. sex, intake of wild forest foodstuff, and alcohol consumption need to be assessed too. Line 175-180 gives the details, but found none in abstract

**a)**	-However, the proportion of a higher number of GI findings increased with the age of the groups. This tendency can be seen in the proportions of the 3 and 4 GI findings. However, the proportion of 5 GI findings was detected only in two age groups, namely 51–60 years and 61–70 years. We also assessed the significance of the average Bq/kg in different age groups, which turned out to have no significant difference (p=0.461). The highets average Bq/kg was observed in the group of 81-83 years old. However this group may not be representative, as it only containes 6 participants. If this group is excluded, the younger the age, the higher the level of internal radiation exposure.

b)	Factors such as Bq/kg and smoking did not affect the increase of number of GI findings.

c)	age, sex, intake of wild food, and alcohol consumption contribute significantly to the increase in the number of GI findings.

d)	They showed significant differences (p<0.05), suggesting that the increased levels of GI findings in detectable groups are more likely to have originated due to alcohol consumption.

The highlighted inputs above should be included in abstract.

**Response.**

We reflected all the recommendations provided above in the abstract of our manuscript concisely. Though the guidelines of PLOS ONE for abstract put some limitations on the number of words and structure, as follows:

- Describe the main objective(s) of the study
- Explain how the study was done, including any model organisms used, without methodological detail
- Summarize the most important results and their significance
- Not exceed 300 words

(Line 38-44 of clean copy)

*Consequently, we revealed that the number of GI diagnosis is significantly increased by factors such as sex, intake of wild forest foodstuff, and alcohol consumption. However, the average level of internal exposure of 137Cs and smoking did not relate to the number of GI diagnosis. Thus, the results of multiple regression revealed that alcohol consumption is independently related to the number of GI diagnosis that is most likely accompanied by the intake of wild forest foodstuff. In conclusion, the low-dose internal exposure may not affect the digestive organs of residents living around CNPP.*

**Comment 7.**

Abst, results – “Other factors, like the average level of a becquerel per kilogram of body mass (Bq/kg) and smoking”. Kindly revised the statement. Its confusing. What activity is this? Cs-137? The for the smoking what is the tangible data? how do you compare that?

**Response.**

We added further details for clarity and to facilitate the comprehension of readers.

(Line 40-41 of clean copy)

*However, the average level of internal exposure of ^137^Cs and smoking did not relate to the number of GI diagnosis.*

**Comment 8.** Abst, conclusion – no conclusion have been drawn.

**Response.**

According to your suggestion, we added the conclusion in the abstract.

(Line 41-44 of clean copy)

*Thus, the results of multiple regression revealed that alcohol consumption is independently related to the number of GI diagnosis that is most likely accompanied by the intake of wild forest foodstuff. In conclusion, the low-dose internal exposure may not affect the digestive organs of residents living around CNPP.*

**Comment 9.**

Abst, recommendation - My opinions is that you draw a conclusion from your 2 years of work before suggesting a study that efficient in proving you hypothesis from this study.

**Response.**

We slightly changed the structure of the sentence and believe that it now conveys the meaning better. There are limitations stated in the guidelines of PLOS ONE for the abstract regarding the number of words and conclusion.

(Line 43-44 of clean copy)

*In conclusion, the low-dose internal exposure may not affect the digestive organs of residents living around CNPP.*

**Comment 10.**

Intro, line 55-60 – instead of stating the existence of BEIR VI’s comprehensive report on effect off low dose, it is more appropriate if the author highlighting the BEIR, IAEA, UNSCEAR and WHO findings on Cs-137 contaminated foodstuffs in Chernobyl.

**Response.**

Contaminated foodstuff intake was not the main focus of this study. This study estimated the effects of chronic low internal radiation on the human body’s organs, particularly on the digestive organs, which remains unexplored. Many papers have reported that it may cause some health effects after several decades have passed. Therefore, we needed to cite recent findings regarding low-dose health outcomes reported by prominent reports that are related to our study. We supplemented information with reference to earlier studies that investigated internal exposure in bodies of people from contaminated areas.

(Line 106-116 of clean copy)

*A few studies investigating the presence of radioactivity in the bodies of people living in contaminated areas around CNPP, showed that a substantial percentage of the population—almost 50% in the beginning of the study year—had some level of radiation [12, 13]. A study conducted from 1996 to 2008 found that 513 participants or 0.35% of the study population had an annual internal radiation dose exceeding 1 mSv, which is the dose limit set by the International Commission on Radiological Protection for the general public [12]. Despite our study was conducted from 2009 to 2018, on screening the residents around CNPP for internal radiation, we found fewer residents (53 participants, 0.02%) with higher levels of dose and radiation detected in their bodies. Consequently, there is still uncertainty regarding the effects of chronic low-dose internal radiation and its health outcomes [13].*

**Comment 11.**

Intro, 61-65 – “highest exposed recovery workers”. Kindly check the suitability to include this information because it may be a dominated by major gamma external exposures. The written introductions are lack of focus. The author highlights the “recovery workers” which out of the topic according to this work aims of motivation and title. It is necessary up to these points, the authors only addressing the literature work of investigation of Cs-137/other isotopes intakes by the Chernobyl’s publics.

**Response.**

We removed the part related to the high-exposed recovery workers but retained the part that states the final finding of the BEIR report.

**Comment 12.**

Intro, 66-75 –the whole paragraph just highlighting the finding for the thyroid cases from ingested iodine contaminated food. Can you make some hypothesis of these finding in relation to your work GI findings based for Cs-137 contamination? At least a line stating that increased I-131 thyroid cases after few years of the incident shows that high possibility of unavoidable Cs-137 ingestion through local foodstuffs.

**Response.**

We added this paragraph to provide the general overview and outcomes caused by internal radiation exposure on the health of people living in contaminated areas around Chernobyl. In respond to your comments, we also deleted partially from lines 68 to 73.

**Comment 13.**

Line 76-116 –These para(s) highlighting the BEIR VI findings of non-cancer risks based on LSS & abs data from UNSCEAR reports in your work. Discussing the background is necessary however you include broaden scopes of internal organ non-cancer incidences. This manuscript will be impactful to readers if you narrow the scopes by focusing the literature works/background studies on GI impacts from low dose or Cs-137 ingestions.

**Response.**

We deleted Lines 82–86 and 94–101 as they were less relevant to our study; however, the other paragraphs are related to the topic of our study, since most of the upper GI findings are of non-cancer diseases.

**Comment 14.**

Line 124-126 - The statement needs citations.

**Response.**

These mentioned suspicions that internal radiation causes GI diseases were expressed verbally by the doctors from the Medical Centre, while we worked on another joint research with them. They pointed out that people from contaminated areas seemingly have relatively higher incidence of GI disease than people in other areas according to their observation and suggested investigating whether the presence of internal radiation possibly causes an increase in GI findings. There is no specific paper to cite.

**Comment 15.**

Line 163-164 - *We used the number of GI findings detected in each participant to indicate the extent of gastrointestinal lesions.* What number? A score? How do you scale it? 1 until 5? please elaborate after the line.

**Response.**

We changed the mentioned sentence as follows and added more details for better comprehension.

(Line 166-170 of clean copy)

*Upper GI endoscopic findings in participants were diagnosed by professional gastroenterologists according to the ICD of WHO. We used the number of upper GI endoscopic diagnosis per person to indicate the extent of GI damages. The number of upper GI endoscopic diagnosis detected in one participant in our dataset varied from 0 to 5 diagnoses.*

**Comment 16.**

Line 163 – Please elaborate the criteria for finding 1 – 5? Extent of lesion and condition?

**Response.**

We changed the mentioned sentence as follows and added more details to improve comprehension.

(Line 166-172 of clean copy)

*Upper GI endoscopic findings in participants were diagnosed by professional gastroenterologists according to the ICD of WHO. We used the number of upper GI endoscopic diagnosis per person to indicate the extent of GI damages. The number of upper GI endoscopic diagnosis detected in one participant in our dataset varied from 0 to 5 diagnoses. Each participant could have various combinations of upper GI diseases, such as gastritis, duodenitis, duodenogastric reflux, gastroesophageal reflux, stomach ulcer, diaphragmatic hernia, and so on.*

**Comment 17.**

Line 166 – radiation? Or activity levels?

**Response.**

We added the word “radioactivity” for clarity.

(Line 163-164 of clean copy)

*The minimum detectable radioactivity level of ^137^Cs on this WBC was 270 Bq per body.*

**Comment 18.**

Line 170 – cleaned? Filtered?

**Response.**

We supplemented that all the data was cleaned and filtered, as shown below.

(Line 181-183 of clean copy)

*All the data was cleaned, filtered and grouped by certain characteristics, such as sex, age, number of detected upper GI endoscopic diagnosis, wild forest food and alcohol intake, and smoking habits.*

**Comment 19.**

Methodology – Please include the work flowcharts of your work. From fill form, wbc screening – GI endoscopy, classification 0 Bq or higher, data treatment, statistical test etc.

**Response.**

We amended the sentences to include details of the workflow to enable simple understanding.

(Line 144-159 of clean copy)

*We invited all patients who sought medical assistance in the Medical Center for any upper GI symptoms or digestive organ disorders that required GI endoscopic intervention during the study period. Residency registration within the research area at the moment of examination was mandatory. Those who agreed to participate in the study, initially received a detailed description of the process and content of the study and were then asked to provide written consent. Afterward, they first completed a questionnaire regarding their lifestyle and dietary habits. Questionnaires were distributed in hard copies and prepared in the Russian language. It consisted of four pages in A4 size and included the respondent’s name, address, date of birth, informed consent, milk and forest food intake, alcohol consumption, smoking habits and their frequency. Once they finished filling the questionnaire, they were invited to udergo upper GI endoscopy and measurement of their internal body burden on the Whole-Body Counter (WBC). All the data collected from the questionnaires, GI endoscopic examinations, and WBC measurements were then used to assess their effects of internal exposure on the upper GI endoscopic findings and identify their associations and contributions.*

(Line 185-192 of clean copy)

*When the internal exposure of the participants was below detectable levels, they were considered and qualified as “0 Bq.” The relevant and necessary statistical tests were conducted and represented in the appropriate way. All statistical analyses were performed on IBM SPSS Statistics 25.0 software. The Mann-Whitney U test and Chi-square tests were used for statistical significance and the determination of averages and proportions. We also ran correlation tests and univariate regression analysis to test the contributions of several variables. P-values lower than 0.05 were considered significant.*

**Comment 20.**

Table 1 - P-value of what significance test? Men vs women?

**Response.**

The P-value in Table 1 represents the significance in the values for men and women. We added an annotation under Table 1.

(Line 222 of clean copy)

*P-value represents the significance in values of men and women.*

**Comment 21.**

Table 2 – Define body burden and detected subject

**Response.**

We deleted the term “body burden” from this paragraph as it may cause some confusion in understanding it; nonetheless, the meanings of body burden and internal exposure are the same throughout our paper. We added the following as annotations under Table 2.

(Line 232 of clean copy)

*Body burden is the value of radioactivity in participants represented in Bq/kg.*

**Comment 22.**

– Based on highlighted lines below, kindly explain how do you treat the difference sizes of population groups in the Mann Whitney U test? How to assure no bias output from the analysis? P=0.461 value may be affected by the significant difference of group size?

We also assessed the significance of the average Bq/kg in different age groups, which turned out to have no significant difference (p=0.461). The highets average Bq/kg was observed in the group of 81-83 years old.

**Response.**

For the analysis mentioned above, we used Tamhane test, which tests the mean (Avg.Bq/kg in each group) difference in significancy compared to other groups.

The distribution of the dataset for all age categories, avg. Bq/kg, and standard deviation are presented in the following table. The group with the fewest participants is the 80-years-old age group, which consisted of only 6 persons. Other groups were distributed relatively evenly. Although the 10-year age group has 53 participants, the number of participants is large enough. The number of participants in each age-group is sufficient to conclude that there is no bias (except in the 80-years group). Additionally, considering their standard deviation, they do not fluctuate into a large extent, which, to us, means that the data are not biased. We believe that the data of avg.Bq/kg. for age groups is fair and not biased (except the 80-years group).

| **Age groups** | **Avg.Bq/kg** | **No. participants** | **SD** |
| --- | --- | --- | --- |
| 10 - years | 6.9 | 53 | 11.7 |
| 20 - years | 5.9 | 224 | 8.2 |
| 30 - years | 6.8 | 215 | 10.6 |
| 40 - years | 6.2 | 276 | 8.8 |
| 50 - years | 6.6 | 380 | 11.6 |
| 60 - years | 6.2 | 346 | 8.7 |
| 70 - years | 4 | 112 | 10.1 |
| 80 - years | 12.5 | 6 | 14.5 |

For more clarity, we added more details into the mentioned paragraph about the number of participants in the groups and the test used for identification of the significance of average Bq/kg between groups.

(Line 272-278 of clean copy)

*We also assessed the significance of the average Bq/kg between age groups using ANOVA test. The ANOVA multiple Comparisons Tamhane test showed no significant difference between age groups (p=0.461). The highest average Bq/kg was observed in the group of 81-83 years old. However, this group may not be representative, as it only contains 6 participants, while all other groups consist of 53-380 participants. If the age group of 81-83 years is excluded, then the younger the age, the higher the level of internal radiation exposure.*

**Comment 23.**

Line 279 - dependent variables? Not independent?

**Response.**

Your remark is correct; thus, we revised the sentence accordingly.

(Line 306-307 of clean copy)

*This analysis evaluates whether these independent variables affect the increase in the number of upper GI endoscopic diagnosis in individuals.*

**Comment 24.**

Line 296-326 – redundant as intro and methodology. Please remove. Discussion is only to discuss your inputs and highlight the finding which started at line 327.

**Response.**

We find the first paragraph necessary for gaining a general understanding of our paper, and we are certain that it is relevant to our study’s topic. Nevertheless, we decided to remove the second paragraph, lines 317-331.

With the incorporation of these amendments to our final manuscript, we hereby resubmit our manuscript for a third evaluation. We hope that the revised version of our paper is now suitable for publication in *PLoS ONE*, and we look forward to hearing from you at your earliest convenience.

Sincerely,

Naomi Hayashida, M.D., PhD.

Professor of Division of Strategic Collaborative Research, Center for Promotion of Collaborative Research on Radiation and Environment Health Effects, Atomic Bomb Disease Institute, Nagasaki University

1-12-4 Sakamoto, Nagasaki 852-8523, JAPAN

TEL: +81-95-819-8507

FAX: +81-95-819-8508

E-mail: [naomin@nagasaki-u.ac.jp](mailto:naomin@nagasaki-u.ac.jp)
